# The Simultaneous Determination of Chlorpyrifos–Ethyl and –Methyl with a New Format of Fluorescence-Based Immunochromatographic Assay

**DOI:** 10.3390/bios12111006

**Published:** 2022-11-11

**Authors:** Zi-Hong Xu, Jia Liu, Bin Li, Jun-Kai Wang, Xi Zeng, Zi-Jian Chen, Surat Hongsibsong, Wei Huang, Hong-Tao Lei, Yuan-Ming Sun, Zhen-Lin Xu

**Affiliations:** 1 Guangdong Provincial Key Laboratory of Food Quality and Safety, South China Agricultural University, Guangzhou 510642, China; 2 Guangzhou Institute of Food Inspection, Guangzhou 510410, China; 3 Guangdong Dayuanlvzhou Food Safety Technology Co., Ltd., Guangzhou 510530, China; 4 School of Health Sciences Research, Research Institute for Health Science, Chiang Mai University, Chiang Mai 50200, Thailand

**Keywords:** chlorpyrifos, hapten design, monoclonal antibody, fluorescence-based immunochromatographic assay, quantitative detection

## Abstract

The improper and excessive use in agriculture of chlorpyrifos–methyl (CPSM) and chlorpyrifos–ethyl (CPSE) may affect the health of human beings. Herein, a fluorescence-based immunochromatographic assay (FICA) was developed for the simultaneous determination of CPSM and CPSE. A monoclonal antibody (mAb) with equal recognition of CPSM and CPSE was generated by the careful designing of haptens and screening of hybridoma cells. Instead of labeling fluorescence with mAb, the probe was labeled with goat-anti-mouse IgG (GAM-IgG) and pre-incubated with mAb in the sample. The complex could compete with CPS by coating antigen in the test line. The new format of FICA used goat-anti-rabbit IgG (GAR-IgG) conjugated with rabbit IgG labeled with fluorescence microspheres as an independent quality control line (C line). The novel strategy significantly reduced nonspecific reactions and increased assay sensitivity. Under the optimal conditions, the proposed FICA showed a linear range of 0.015–64 mg/L and limit of detection (LOD) of 0.015 mg/L for both CPSE and CPSM. The average recoveries of CPS from spiked food samples by FICA were 82.0–110.0%. The accuracy was similar to the gas chromatography–tandem mass spectrometry (GC-MS/MS) results. The developed FICA was an ideal on-site tool for rapid screening of CPS residues in foods.

## 1. Introduction

Chlorpyrifos (CPS), including chlorpyrifos–methyl (CPSM) and chlorpyrifos–ethyl (CPSE), is widely used in agricultural production for the control of aphid, bollworm, leafhopper, mite, and other pests [1,2]. As a class II moderate-toxicity pesticide (according to the WHO classification), CPS is in up to 1/3 of all conventionally produced citrus fruits [3]. CPS that accumulates in food can enter the human body through the food chain. It can form a stable complex with the active site of acetylcholinesterase and prevent acetylcholinesterase as a catalyst, which will result in an adverse effect on the nervous system. CPS can also induce endocrine disruption, cardiovascular diseases, developmental and behavioral anomalies, hematological malignancies, genotoxicity, histopathological aberrations, immunotoxicity, and oxidative stress [4]. The maximum residue limits (MRLs) for CPS in food products have been stipulated, including 0.01–20 mg/kg in China [5] and 0.01–6 mg/kg in Europe [6]. Moreover, the United States has prohibited its use since 2018. However, it is still in use in developing countries because of its persistence and effectiveness in residual form [7]. Therefore, it is of great significance to establish effective methods for CPS detection.

Several analytical methods for CPS detection have been reported, including high-performance liquid chromatography [8,9], liquid chromatography–mass spectrometry [10,11], and immunoassay. Compared with traditional instrumental methods, the antibody-based immunoassay has developed rapidly in recent years due to its low cost and high throughput. At present, ELISA and gold immunochromatographic assay (GICA) are the two most popular immunoassays for pesticide screening, including CPS. Maftouh et al. developed an ELISA using polyclonal rabbit antibodies to detect CPSE and its two significant degradation metabolites [12]. However, the problems with ELISA lie mainly in the multistep detection process and long testing time. Kim et al. established a GICA based on a competitive antigen-coated format to detect CPSE [13]. Hua et al. developed a GICA to rapidly detect CPSM in water samples [14]. GICA is based on the reaction of the colloidal gold particles labeled antibody and antigen to cause chromogenic reaction. It is an ideal screening tool for on-site pesticide residue detection in food samples due to its rapidity, low cost, and absence of instruments [15]. However, most of the reported immunoassays for CPS detection are highly specific to CPSE or CPSM [14,16,17]. The simultaneous detection of CPSE and CPSM is necessary for improving the determination efficiency. Moreover, the colloidal gold-based GICA for pesticide screening is qualitative or semi-quantitative, leading to false positives for those pesticides with MRLs. In order to improve the sensitivity of the GICA, much effort and cost were spent on developing new probes. The fluorescence microsphere is one of the particularly attractive labeling probes and has received great expectations regarding the sensitive and quantitative detection of trace analytes since it effectively improved the sensitivity of quantitative detection and retained the advantages of convenience, rapidness, and simplicity of the typical strip [18]. Cheng et al. developed an ICA based on a fluorescence microsphere for the accurate quantitative detection of ochratoxin A in rice flour [19]. Zhou et al. established a fluorescence-based immunochromatographic assay (FICA) to detect deoxynivalenol in agricultural products, showing that FICA represents a good strategy for food safety detection [20]. In addition, the other alternative for increasing the sensitivity of competitive ICA system is reducing the amount of specific monoclonal antibody (mAb). However, the traditional labeling strategy is to label the fluorescence microspheres directly on the mAb, which leads to the synchronous reduction of mAb and fluorescence microspheres. Thereby, the reduced amount of antibody directly caused the signal intensity decrease in the test line (T Line) and hampered a successful detection process [21]. The indirect labeling of mAb in which goat-anti-mouse IgG (GAM-IgG) specific to mAb is labeled with fluorescence can overcome the limitations of conventional labeling since the mAb contains several epitopes that can bind to labeled secondary antibodies, allowing signal amplification [22].

In this study, we aim to obtain a mAb with high sensitivity by carefully designing haptens and screening hybridoma cells. Firstly, two novel haptens were synthesized by replacing o-ethyl at the thiophosphate moiety with -Cl and -NH(CH_2_)_5_COOH in the CPSE structure, respectively. Artificial antigens were synthesized and then used in animal immunization based on these two haptens. A mAb with equal recognition to CPSM and CPSE was obtained by the careful screening of hybridoma. Then, the FICA for CPS screening in fruits and vegetables was developed. In order to override the nonspecific reactions and increase the test strip sensitivity, we attempted to develop the FICA through the combination of two techniques including: (1) indirect labeling whereby the fluorescence probe was labeled with GAM-IgG and pre-incubated with mAb instead of labeling fluorescence with mAb; (2) developed a new format of FICA using goat-anti-rabbit IgG (GAR-IgG) labeled with fluorescence microspheres reacted with rabbit IgG as an independent control line (C Line) and simultaneously detected CPSM and CPSE, which has not been reported so far. The performance of FICA was validated by specificity and recovery rate, and the results were validated by a standard GC-MS/MS.

## 2. Materials and Methods

### 2.1. Chemicals and Reagents

The chemicals CPSM and CPSE (the purity ≥ 99.0% by HPLC) were purchased from the Agro-Environmental Protection Institute, Ministry of Agriculture and Rural Affairs (Tianjin, China). Fluorescence microsphere, lactoferrin (LF), bovine serum protein (BSA), 1-ethyl-3-(3-dimethylaminopropyl) carbodiimide (EDC), and N-hydroxysuccinimide (NHS) were purchased from Sigma-Aldrich, Inc. (St. Louis, MO, USA). GAM-IgG, rabbit IgG, and GAR-IgG were purchased from TransGen Biotech (Beijing, China). Nitrocellulose (NC) membrane, absorption pad, and sample pad were purchased from Jieyi Co., Ltd. (Shanghai, China). The protein G resins were obtained from TransGen Biotech Co., Ltd. (Beijing, China). The water used in the experiment was ultrapure, and all other chemical reagents were analytical grade and purchased from Guangzhou Chemical Reagent Factory (Guangzhou, China).

### 2.2. Apparatus

The QP50 mass spectrometer was provided by Shimadzu International Trading Co., Ltd. (Shanghai, China). The AVANCE 500 MHz nuclear magnetic resonance apparatus was purchased from Brock (Ettlingen, Germany). The Wellwasher and the Multiskan™ FC were purchased from Thermo Fisher Scientific (Waltham, MA, USA). The GY-706 fluorescence quantitative detector was supplied by Henan Guanyu Instrument Co., Ltd. (Zhengzhou, China). The HGS510 cross membrane system and the HGS201 cutting system were provided by Hangzhou Fenghang Technology Co., Ltd. (Hangzhou, China). Chromatographic analysis was performed using the Agilent 8890-7000D gas chromatography-tandem triple quadrupole mass spectrometer (Agilent Technologies, Waldbronn, Germany) equipped with a Thermo TR-PESTICIDE Ⅱ column supplied by Thermo Fisher (Shanghai, China). The MassHunter WorkStation Acquisition Software rev.B.02.01 was supplied by Agilent Technologies (Waldbronn, Germany).

### 2.3. Synthesis of Haptens and Artificial Antigens

**Figure 1:**
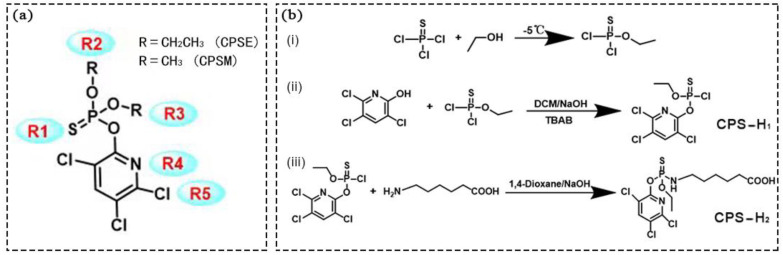
(**a**) Structure of CPS. (**b**) Synthetic route for hapten CPS-H_1_ and hapten CPS-H_2_. (i) The synthesis process of O-methyl thiophosphoryl dichloride. (ii) The synthesis process of hapten CPS-H_1_. (iii) The synthesis process of hapten CPS-H_2_.

Synthesis and characterization of haptens: as shown in Figure 1a, there were five prospective positions on CPS for the introduction of linkers, viz. sulphur (R1) and o-ethyl/o-methyl (R2 and R3) in thiophosphate group, nitrogen (R4) and 6th chlorine (R5) in the pyridyl ring [23]. In this study, the haptens were designed according to our previous work [24]: the o-ethyl at the thiophosphate moiety of CPSE was replaced with -Cl and -NH(CH_2_)_5_COOH for the synthesis of haptens (Figure 1b).

Synthesis of O-methyl thiophosphoryl dichloride: The phosphorous trichloride (55 mL) was added to a 200 mL round-bottomed flask with stirring at −5 °C. Afterward, ethanol (100 mL) was added slowly, and the reaction was allowed to proceed for 6 h. The mixture was slowly and carefully washed with ice water twice to remove excess ethanol. The purified product was storied at 4 °C.

Synthesis of hapten CPS-H_1_: The 3,5,6-trichloropyridine-2-alcohol (3.6 g), O-methyl thiophosphoryl dichloride (3 g) and tetrabutylammonium bromide (0.5 g) were dissolved in 10 mL dichloromethane and the mixture was stirred overnight. Then the NaOH aqueous solution (10 mL, 50 mg/mL) was added into the above mixture under vigorous stirring overnight to ensure the disappearance of the organic-water phase stratification interface. After the reaction ended and the mixture was stratified, the crude product was obtained by rotary evaporation from the organic phase. The hapten CPS-H_1_ was obtained by purifying the crude product with 400 mesh silica gel chromatography (mobile phase = ethyl acetate: petroleum ether = 1:10). The mass and ^1^H NMR spectrogram of the hapten CPS-H_1_ in Figures S1 and S2 showed that the hapten was synthesized successfully. ESI-MS (positive) m/z: 342 [M + H]^+^, ^1^H NMR (600 MHz, MeOD) δ 8.26 (d, J = 1.0 Hz, 1H), 4.39 (m, 2H), 1.41 (m, 3H).

Synthesis of hapten CPS-H_2_: CPS-H_1_ (0.5 g), 6-aminocaproic acid (5 g) and 1,4-dioxane (10 mL) were added into a round-bottom flask with stirring at low temperature. Then the NaOH solution (20 mL) was added to the above mixture to maintain pH 8–9 overnight, and the pH of the solution was adjusted to 4–5 with HCl. The crude product in the aqueous phase was extracted by ethyl acetate, and the final product hapten CPS-H_2_ was obtained by rotary evaporation. The results of mass and ^1^H NMR spectrogram of the hapten CPS-H_2_ are shown in Figures S3 and S4 shows that the hapten has been synthesized successfully. ESI-MS (negative) m/z: 434 [M-H]^−^, ^1^H NMR (600 MHz, MeOD) δ 8.16 (s, 1H), 4.30 (dd, J = 22.4, 7.1 Hz, 2H), 3.13 (dd, J = 12.9, 6.4 Hz, 2H), 2.28 (t, J = 7.5 Hz, 2H), 1.61 (m, 4H), 1.40 (dt, J = 14.2, 7.2 Hz, 5H).

Synthesis and characterization of artificial antigens: CPS-H_1_ was directly conjugated with ε-amino in BSA. The CPS-H_1_ was dissolved in dioxane and dropwise added to BSA (dissolved in carbonate buffer) to couple overnight at room temperature. CPS-H_2_ was conjugated to LF or BSA for artificial antigens by the carbodiimide method according to previous work with some modifications [25]. Briefly, the CPS-H_2_ was dissolved in DMF, and activated by EDC and NHS at room temperature and stirred at 4 h. Then the above activation solutions were added to LF and BSA (dissolved in carbonate buffer) to couple overnight at room temperature. Finally, all the above conjugates were dialyzed with phosphate buffer for 3 days. The purified coating antigen CPS-H_1_-BSA, CPS-H_2_-BSA and immunogen CPS-H_2_-LF were obtained. The UV-vis spectra showed that the characteristic peaks of haptens had significant changes after conjugating with the carrier protein (Figure S5), indicating that the artificial antigens were successfully synthesized.

### 2.4. Preparation and Characterization of Anti-CPS mAb

The animal immunization procedure was described in our previous study [25]. The female Bal b/c mice were fed at the South China Agriculture University Animal Centre. All the animal experiments were performed in compliance with the relevant protective and administrative laws for laboratory animals of China and conducted with the approval of Institutional Authority for Laboratory Animal Care (ethical approval number: 2021B090). The mouse that exhibited great immune response was chosen as the donor of spleen cells for hybridoma production.

Cell lines secreting mAb against CPS were obtained according to previously described methodology [25]. The hybridoma cell lines were screened, which can recognize CPSE through five rounds limiting dilution. The obtained mAb was purified by commercialized protein G resin. Then the sensitivity and specificity of mAb were determined. The sensitivity test was evaluated as the half maximal inhibitory concentration (IC_50_) of mAb to CPSE. The specificity of mAb was assessed by cross-reactivity (CR) between several related compounds with mAb. Therefore, chlorpyrifos–methyl, parathion, fensulfothion, carbofuran, triazophos, parathion–methyl, coumaphos, quintiofos, and 3,5,6-Trichloro-2-pyridinol were selected as inhibitors under the same experimental conditions. The CR was calculated on the basis of IC_50_ with following formula: $\mathrm{CR}\;=\left[{\mathrm{IC}}_{50}\;\left(\mathrm{CPSE}\right)/{\mathrm{IC}}_{50}\;\left(\mathrm{analog}\right)\right]\times 100\;\%$ [26]. Where IC_50_ (CPSE) is the IC_50_ of mAb to CPSE, and IC_50_ (analog) is the IC_50_ of mAb to CPSE analog.

### 2.5. Preparation of Fluorescence Probes

The method of preparation of fluorescence probes was modified according to the reported studies [2]. Scanning electron microscopy revealed that the fluorescence microsphere was homogeneous with a particle size of approximately 200 nm (Figure S6)**.** In order to activate the fluorescence microsphere carboxyl groups, a total of 1 mL of the fluorescence microspheres solution with 10% concentration was added to 100 μL NHS (100 mg/mL) and 100 μL EDC (100 mg/mL) successively and activated at room temperature for 1 h. After being centrifuged and redissolved with MES buffer (pH 8), a certain amount of GAM-IgG (1 mg) and GAR-IgG (0.25 mg) was added to the above solution, respectively. The mixture was vortexed for 3 h before adding 100 μL of 10% BSA, and then vortexed for another 1 h. The mixture was centrifuged at 7602× *g* for 10 min. The ultrapure water was used to wash precipitate three times and resuspended in 1 mL fluorescence probe dilution. Finally, the fluorescence microsphere labeled GAM-IgG probe (10 µL), fluorescence microsphere labeled GAR-IgG probe (1 µL), and anti-CPS mAb (0.05 mg) were added into the fluorescence probe dilution (1 mL) to prepare the fluorescence solution.

### 2.6. Development of the FICA

**Scheme 1:**
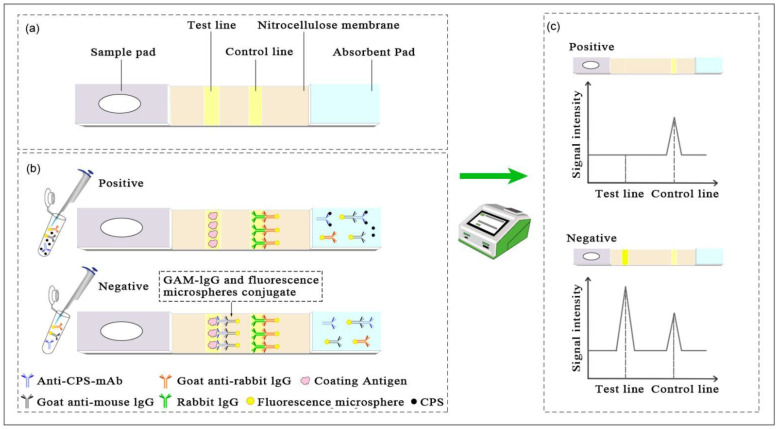
Schematic illustration of the FICA detection process of CPS. (**a**) The composition of FICA. (**b**) The detection process of FICA. (**c**) The detection results of FICA.

The FICA strip was prepared according to the method of Liang et al. [27]. As illustrated in Scheme 1, the FICA mainly consists of polyvinyl chloride backing pad, absorbent pad, nitrocellulose membrane, and sample pad. The T Line and C Line were drawn with CPS-H_1_-BSA (500 mg/L) and the rabbit IgG (300 mg/L), respectively, and dried at 45 °C for 12 h under vacuum. Then the sample pad and absorbent pad were pasted. Finally, the strip was placed in a sealed plastic bag for use.

The principle of the FICA is mainly based on the indirect competitive immunological mode between specific antigen and antibody. Scheme 1 presents the detection process. For the negative samples, anti-CPS mAb bind to GAM-IgG labeled with fluorescence microspheres were captured by CPS-H_1_-BSA on the T line and the GAR-IgG labeled with fluorescence microspheres were captured by rabbit IgG on the C line. For the positive samples, free CPS competes with CPS-H_1_-BSA to capture the anti-CPS mAb, which results in the T-line color shallowing. Since there is no competition between the T line and C line, the color of the C line remains unchanged.

### 2.7. Optimization of FICA

To improve the performance of FICA for CPS, several parameters were optimized, including the optimal labeling pH, the concentrations of antibody and antigen, the CPS standard buffer (pH and ion concentration), the fluorescence probe dilution (pH, Tween-20, sucrose and BSA), and the sample pad pretreatment solution (pH, Tween-20, sucrose and BSA). According to the fluorescence intensity of the T line and C line of the strip tested with 0.2 M phosphate buffer (PB) or positive (1 mg/L CPSE) solution, the parameter with the strongest fluorescence intensity and obvious inhibition effect was selected as optimal [28]. The calibration curve was constructed as follows: CPS was serially diluted with 0.2 M PB and tested by FICA. Then the calibration curve was generated using Origin pro 7.5 software (OriginLab Corp., Northampton, MA, USA) with the T/C was the ordinate and the log of CPSE/CPSM concentration was the abscissa.

### 2.8. Sample Preparation

CPS is an insecticide commonly used in vegetable and fruit production. The Chinese cabbage, lettuce, pear, and apple, which are commonly consumed in China, were evaluated. The blank samples of vegetable and fruit were purchased from the local market in Guangzhou. The fruit samples were pretreated as follows: a total of 2 g of the homogenized fruit samples (pear and apple) was weighed, and three concentrations of CPS (1.0, 5.0, and 25.0 mg/kg) were spiked into the samples. Subsequently, 8 mL of sample extraction (PB, 0.2 mol/L, pH 8) was added to mix thoroughly. The extracting solution was shifted for use. The maximum residue level of CPS on vegetable samples is stricter [5,6]. To improve the extraction effect, the vegetable samples’ pretreatment was optimized by the extraction solution and extraction temperature. The final preparation steps were described as follows: the homogenized samples (2 g) was added into a 10 mL centrifuge tube. Three concentrations of CPS (0.1, 0.5, and 2.5 mg/kg) were spiked into the vegetable samples (Chinese cabbage and lettuce). Then anhydrous sodium sulfate (0.5 g) and 0.5% trichloroacetic acid in acetonitrile (0.5% TCAA-MeCN) (2 mL) were added. The mixtures were shaken for 1 min and centrifuged at 2325× *g* for 3 min. Then the organic layer (1 mL) was taken out for blow-drying at 30 °C. Finally, a volume of 4 mL PB buffer (0.2 mol/L, pH 8) was added to re-dissolve the residue for use. The matrix effect of the samples can be removed by 4-fold dilution. After sample pretreatment, a volume of 40 μL of the sample extract was mixed with the fluorescence solution in equal volume and submitted for FICA analysis. The fluorescence intensity of the T line and C line were read after 10 min, and the recovery rate was calculated.

### 2.9. Validation by GC-MS/MS

The accuracy of the proposed FICA was estimated by recovery test and confirmed by reference methods (GC-MS/MS) performed with reference to Chinese National Standards [29]. Chromatographic analysis was performed using the Agilent 8890-7000D gas chromatography–tandem triple quadrupole mass spectrometer. Chromatographic resolution was achieved using a Thermo TR-PESTICIDE Ⅱ column (30 m × 0.25 mm I.D., 0.25 μm film thickness). The initial oven temperature was 60 °C (maintained for 0.5 min) then increased to 280 °C at 20 °C/min (maintained for 5.5 min). The injection inlet temperature was 270 °C and the detector temperature was set at 280 °C. The sample was injected into the gas chromatograph by splitless injection mode with a constant flow rate of 1.15 mL/min of the helium flow (carrier gas). The mass spectrometer was operated where the nitrogen (carrier gas) constant flow rate of 1.5 mL/min. Data were collected in the MRM mode, using the MassHunter WorkStation Acquisition Software rev.B.02.01.

## 3. Results and Discussion

### 3.1. Screening and Characterization of Anti-CPS mAb

**Figure 2:**
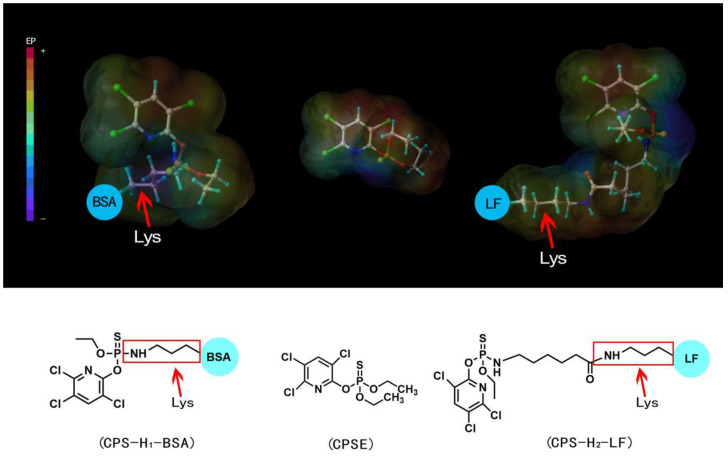
The lowest energy conformations and molecular electrostatic potential isosurfaces of CPSE and haptens.

Haptens design is the key point of immunoassay development and determines the sensitivity and specificity of antibody [30]. Previous studies mostly used the method of introducing NH(CH_2_)_n_COOH to the thiophosphate group of the CPS [12,31,32,33]. Existing examples suggest that the most intact immunogens are likely to produce effective antibodies. In addition, the haptens used for the synthesis of coated antigen should have a close structural similarity with CPS to improve the specificity of the mAb [23]. Thus, we introduced the binding site from the thiophosphate group to prepare the intermediate without spacer arm. It can directly prepare the coating antigen, which saves time and cost. Then, the -NH(CH_2_)_5_COOH group was introduced to the intermediate as an immunogen to fully expose the characteristic group to obtain effective antibodies. The minimum energy conformations of CPSE and haptens are optimized to predict the rationality of hapten design, and the results are shown in Figure 2. Generally, the hapten that was most similar to the target analyte in terms of structure and electrons exhibited high sensitivity and specificity to the target analyte [30]. The following theoretical considerations were made regarding hapten design: Replacing o-ethyl at the thiophosphate moiety by -Cl and -NH(CH_2_)_5_COOH in the CPSE structure. The trichloropyridine ring structure and the thiophosphate group as important epitopes are preserved. The CPS-H_1_ has a short spacer arm that has high steric hindrance and is difficult to interact with antibodies, so it can reduce the competitiveness of antibodies and improve the inhibition rate of CPSE. Apart from that, the electronic effect of CPS-H_1_-BSA is similar to CPSE. The CPS-H_2_ contains long spacer arms (5-carboxylic acids) that can fully expose antigenic determinants and enhance the specific binding of antigen and antibody. Therefore, CPS-H_1_-BSA was used as a coating antigen and CPS-H_2_-LF was used as an immunogen.

**Figure 3:**
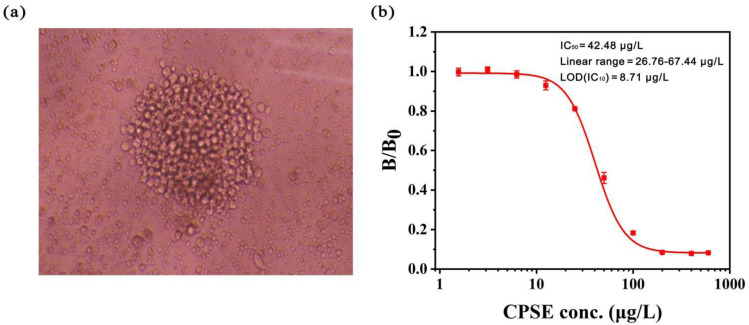
(**a**) The morphology of cells after fusion. (**b**) Calibration curve for determination of CPSE.

CPS-H_2_-LF was used as an immunogen to immunize mice and ic-ELISA was performed to determine the titer and inhibition of antiserum. Table S1 showed that the antiserum exhibited higher titer using CPS-H_2_-BSA as a coating antigen, whereas its inhibition was significantly decreased. When CPS-H_1_-BSA was used as a coating antigen, it exhibited good inhibition rate, indicating that antibody against CPSE was produced in serum. The long spacer arms of CPS-H_2_ promote exposure to antigenic determinants, which enhances antibody binding to CPS-H_2_ and reduces the inhibition of CPSE. Therefore, homologous coating CPS-H_2_-BSA is not suitable for establishing a highly sensitive immunoassay for CPSE. Then spleen cells were fused with SP2/0 myeloma cells, and positive cell lines were screened. Finally, a single cell line (4E6) secreting anti-CPS mAb was obtained. Figure 3a was the morphology of cells after fusion. As shown in Figure 3b, the ic-ELISA showed linear range of 26.76–67.44 μg/L and limit of detection (LOD) was 8.71 μg/L, with an IC_50_ value of 42.48 μg/L for CPSE. The specificity of anti-CPS mAb is showed in Table 1 No obvious CR was observed with any of the related compounds except CPSM with the highest CR value (CR = 103.89%), which possesses the same thiophosphate group and pyridyl ring structure as the CPSE. In the absence of thiophosphate group, the recognition of the anti-CPS mAb is significantly decrease, such as 3,5,6-Trichloro-2-pyridinol (CR < 0.50%), suggesting that the specific recognition site of the antibody is on the thiophosphate group of the immunogen. Thus, the obtained mAb can both recognize CPSE and CPSM.

### 3.2. Development and Optimization of FICA

Compared with previous reports [13,34], the anti-CPS mAb obtained in this study showed lower sensitivity. In order to reduce nonspecific reactions and increase the sensitivity of quantitative determination for CPS, we developed a novel labeling strategy that the fluorescence probe was labeled with GAM-IgG and pre-incubated with mAb in the sample.

Several physicochemical parameters were then optimized to obtain optimal assay performance of the established FICA. The pH of the labeling reaction system greatly affects the sensitivity, stability, and fluorescence intensity of ICA [2]. As illustrated in Figure S7a, with the increasing pH of the MES buffer, the T/C and inhibition ratio increased to the peak at pH 8. However, the extreme pH adversely affected the fluorescence intensity. The MES buffer at pH 8 was chosen as the most suitable value for the subsequent studies. The amount of antibody and antigen play a key role in the signal intensity and inhibition effect of the strip. As shown in Figure S7b, the optimal concentration of mAb and antigen was 0.05 mg/L and 500 mg/L, respectively. Similarly, as shown in Figure S7c,d, when the pH and the ion concentration of the CPS standard buffer was 8 and 0.2 mol/L, respectively, the strip’s inhibition rate was highest. The parameters of fluorescence probe dilution (pH, BSA, sucrose and Tween-20 concentration) were then optimized for improving the sensitivity and stability of FICA (Figure S8a–d). When the fluorescence probe dilution was at pH 7 and contained 2% BSA, 1% sucrose, and 0.2% Tween-20, the inhibition rate reached the highest with a relatively high signal intensity. Simultaneously, several parameters of the sample pad pretreatment solution were optimized (Figure S9a–d). The final pretreatment solution for sample pad was set at pH 6, containing of 0.5% sucrose, 0.1% Tween-20, and 1% BSA. The developed FICA demonstrated the best performance under this condition.

### 3.3. Sample Pretreatment

In order to improve the extraction effect of vegetable samples, this study optimized the extraction solution and extraction temperature of the pretreatment. The extraction rates of ethyl acetate (EtOAc), methanol (MeOH), acetone (BLE), MeCN, and 0.5% TCAA-MeCN were compared, and the extraction effects were also compared at 30 °C, 40 °C, 50 °C, and 60 °C. As shown in Figure S10a, the 0.5% TCAA-MeCN was the most suitable extraction solution with an applicable recovery rate. Figure S10b illustrates that the recovery rate decreases with the increase in temperature, and the recovery rate is the highest at 30 °C. The reason is that rising temperatures can partially break down the CPS [35]. We determined that the extraction solution of the sample was 0.5% TCAA-MeCN and the extraction temperature was 30 °C.

### 3.4. Evaluation of the FICA

**Figure 4:**
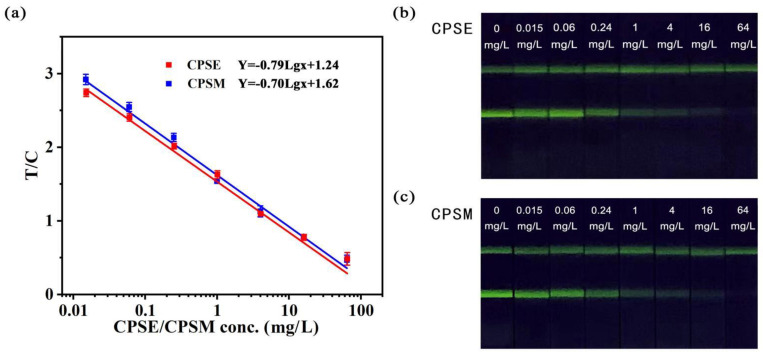
(**a**) Calibration curve of FICA for CPSE and CPSM. (**b**) The test results of serial concentration of CPSE standard solution. (**c**) The test results of serial concentration of CPSM standard solution.

Under the optimal conditions, FICA was performed with serial concentrations of CPS standard solution (80 μL/card). As shown in Figure 4, the T/C decreased with an increase in the concentration of CPS. When the T/C was significantly different from that of the blank sample (B_x_ = B_0_ − 3SD), the target concentration was defined as LOD [36]. Thus, the calibration curve showed the LOD was 0.015 mg/L, and had a linear range of 0.015–64 mg/L. The linear equations of the CPSE, CPSM were Y = −0.78812LgX + 1.2384 (R^2^ = 0.974), and Y = −0.70015LgX + 1.62137 (R^2^ = 0.993), respectively. The immunoassays for the determination of CPS in recent years are summarized in Table 2. This assay detected both CPSE and CPSM, whereas the previous study only detected one of them. The sensitivity of this assay was not significantly improved compared with previous reports, but it can meet the maximum residue level requirements for CPS.

Accelerated stability experiments are carried out at high temperatures for short periods, and if no significant changes are observed during the test, the results have excellent stability [39,40]. In this study, the accelerated experiment indicated that the fluorescence liquid stored at 4 °C and 37 °C showed no significant changes for 2, 4, 6, 8, or 10 days (Table S2), and the strip stored at 37 °C and 45 °C also showed no significant changes for 1, 7, or 15 days (Table S3). The results showed that the FICA for CPS had excellent stability. Moreover, the repeatability was evaluated based on intra-assay and inter-assay experiments [41]. The results listed in Tables S4 and S5, the low CVs demonstrated that the FICA could meet the quantitative requirements of the CPS and be applied in actual production.

### 3.5. Comparison of the FICA with GC-MS/MS

The developed FICA was applied to detect CPSE and CPSM in actual samples and compared with GC-MS/MS to assess its effectiveness. Samples (Chinese cabbage, lettuce, pear, and apple) were collected from local supermarkets, and FICA and GC-MS/MS analyzed the four spiked samples. The results are summarized in Table 3. The average recoveries obtained ranged from 82.0% to 110.0% and 80.0% to 106.4% for FICA and GC-MS/MS, respectively. The results indicated that the accuracy of the developed FICA was similar to GC-MS/MS.

## 4. Conclusions

Based on the reasonable designing of haptens and careful screening of hybridoma, the present study obtained the mAb with equal recognition for CPSE and CPSM. A new format of FICA for CPS based on the mAb was developed. Instead of labeling fluorescence with mAb, the probe was labeled with GAM-IgG and pre-incubated with mAb in the sample. The complex can compete with CPS by coating antigen in the test line, and the independent C line was coated with rabbit IgG conjugated with GAR-IgG labeled with fluorescence microspheres. Moreover, the new format of FICA with independent C line significantly reduces nonspecific reactions and increases assay sensitivity by reducing the amount of antibody directly. The proposed FICA showed a linear range of 0.015–64 mg/L, and the LOD was 0.015 mg/L for CPS. The GC-MS/MS method verified the performance of FICA in actual samples, and a similar accuracy was obtained. In summary, the above advantages indicated that the developed FICA was an ideal tool for on-site, rapid, and sensitive screening of CPS residues in food samples.

## Figures and Tables

**Table 1:** Specificity evaluation of mAb.

Compound	Structure	IC_50_ (μg/L)	CR (%)
Chlorpyrifos–ethyl	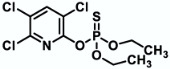	42.48	100.00
Chlorpyrifos–methyl	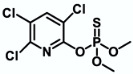	40.89	103.89
Parathion	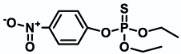	>1000	1.62
Fensulfothion	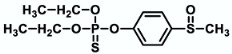	>1000	0.74
Carbofuran	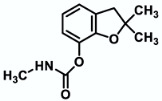	>1000	<0.50
Triazophos	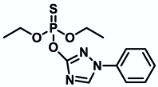	>1000	<0.50
Parathion-methyl	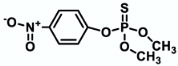	>1000	<0.50
Coumaphos	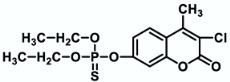	>1000	<0.50
Quintiofos	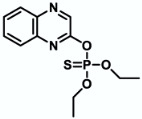	>1000	<0.50
3,5,6-Trichloro-2-pyridinol	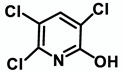	>1000	<0.50

**Table 2:** Comparison of immunoassay for the detection of CPS.

Detection Compound	Signal Tracer	Sample	LOD (mg/L)	Reference
CPSE	Colloidal gold	-	4	[37]
CPSM	Colloidal gold	Water	0.6	[14]
CPSE	Colloidal gold	Rice	0.05	[13]
CPSE	g-C_3_N_4_/BiFeO_3_ nanocomposites	Water	0.000033	[38]
CPSE, CPSM	Fluorescence microsphere	Chinese cabbage, lettuce, pear, apple	0.015	This work

**Table 3:** Recoveries of CPS from spiked food samples by FICA and GC-MS/MS ^a^ (*n* = 3).

CPS	Sample	Spiked Level (mg/kg)	FICA			GC-MS/MS		
			Measured (mg/kg) (M ± SD ^b^)	Recovery ^c^ (%)	CV ^d^ (%)	Measured (mg/kg) (M ± SD)	Recovery (%)	CV (%)
CPSE	Chinese cabbage	0.1	0.11 ± 0.01	110.0	9.1	0.08 ± 0.00	80.0	0.0
		0.5	0.43 ± 0.05	86.0	11.6	0.40 ± 0.00	80.0	0.0
		2.5	2.05 ± 0.06	82.0	2.9	2.25 ± 0.01	90.0	0.4
	Lettuce	0.1	0.11 ± 0.01	110.0	9.1	0.09 ± 0.00	90.0	0.0
		0.5	0.50 ± 0.01	100.0	2.0	0.40 ± 0.00	80.0	0.0
		2.5	2.29 ± 0.04	91.6	1.7	2.26 ± 0.01	90.4	0.4
	Pear	1.0	1.04 ± 0.20	104.0	19.2	0.85 ± 0.01	85.0	1.2
		5.0	5.10 ± 0.10	102.0	2.0	4.60 ± 0.03	92.0	0.7
		25.0	25.26 ± 0.14	101.0	0.6	22.68 ± 0.02	90.7	0.1
	Apple	1.0	1.05 ± 0.03	105.0	2.9	0.88 ± 0.00	88.0	0.0
		5.0	5.18 ± 0.06	103.6	1.2	4.62 ± 0.01	92.4	0.2
		25.0	26.84 ± 0.68	107.4	2.5	22.65 ± 0.01	90.6	0.0
CPSM	Chinese cabbage	0.1	0.10 ± 0.01	100.0	10.0	0.09 ± 0.00	90.0	0.0
		0.5	0.54 ± 0.01	108.0	1.9	0.46 ± 0.02	92.0	4.3
		2.5	2.38 ± 0.10	95.2	4.2	2.40 ± 0.08	96.0	3.3
	Lettuce	0.1	0.11 ± 0.01	110.0	9.0	0.10 ± 0.01	100.0	10.0
		0.5	0.45 ± 0.01	90.0	2.2	0.41 ± 0.01	82.0	2.4
		2.5	2.43 ± 0.05	97.2	2.1	2.48 ± 0.02	99.2	0.8
	Pear	1.0	0.94 ± 0.06	94.0	6.4	0.91 ± 0.00	91.0	0.0
		5.0	4.61 ± 0.17	92.2	3.7	5.18 ± 0.07	103.6	1.4
		25.0	24.13 ± 0.70	96.5	2.9	25.21 ± 0.32	100.8	1.3
	Apple	1.0	1.02 ± 0.02	102.0	2.0	0.91 ± 0.00	91.0	0.0
		5.0	4.70 ± 0.07	94.0	1.5	5.32 ± 0.06	106.4	1.1
		25.0	24.50 ± 0.66	98.0	2.7	25.44 ± 0.22	101.8	0.9

^a^ For one concentration, three positive samples were spiked and determined by FICA and GC-MS/MS. ^b^ SD, standard deviation. ^c^ The samples chosen for the recovery test were negative, which were confirmed and detected by GC-MS/MS. ^d^ CV, coefficient of variance, which was obtained from intra-assay.

## Data Availability

Not applicable.

## Supplementary Materials

The following supporting information can be downloaded at: https://www.mdpi.com/article/10.3390/bios12111006/s1, Figure S1: ESI-MS spectrum (positive) of CPS-H_1_; Figure S2: ^1^H NMR spectrum of CPS-H_1_; Figure S3. ESI-MS spectrum (negative) of CPS-H_2_; Figure S4. ^1^H NMR spectrum of CPS-H_2_; Figure S5. The UV wavelength scanning spectra of artificial antigens. (a) CPS-H_1_-BSA. (b) CPS-H_2_-LF. (c) CPS-H_2_-BSA; Figure S6. The electron microscope scan of fluorescence microspheres; Figure S7. The signal intensity and inhibition results of (a) The labeling pH value; (b) The concentrations of antigen and antibody; (c,d) The pH and ion concentration of the CPSE standard buffer. The spiked target concentrations of the inhibitory effect in Figure S7 (a–d) were 1 µg/mL; Figure S8. The signal intensity and inhibition results of fluorescence probe dilution (a) pH; (b) BSA concentration; (c) Sucrose concentration; (d) Tween-20 concentration. The spiked target concentrations of the inhibitory effect were 1 µg/mL; Figure S9. The signal intensity and inhibition results of sample pad pretreatment solution (a) Sucrose concentration; (b) Tween-20 concentration; (c) BSA concentration; (d) pH. The spiked target concentrations of the inhibitory effect were 1 µg/mL; Figure S10. Optimization of sample pretreatment conditions (n = 3). (a) Extraction solution. (b) Extraction temperature; Table S1. Characterization of the mouse antiserum against free CPSE; Table S2. Stability of fluorescent liquid (n = 3); Table S3. Stability of FICA (n = 3); Table S4. Test results of precision of intra-assay (n = 3); Table S5. Test results of precision of inter-assay (n = 3).
