# Supplementary material for: The Simultaneous Determination of Chlorpyrifos–Ethyl and –Methyl with a New Format of Fluorescence-Based Immunochromatographic Assay
Source: Biosensors (Basel). 2022 Nov 11;12(11):1006. doi: 10.3390/bios12111006 (PMC9688337; doi:10.3390/bios12111006)
Supplement: Supplementary file 1 [file biosensors-12-01006-s001.zip › biosensors-2001784-supplementary.pdf]

Supplemental Information

Simultaneous determination of chlorpyrifos-ethyl and -methyl by new format of fluorescence-based immunochromatographic assay based on a monoclonal antibody

Zi-Hong Xu, Jia Liu, Bin Li, Jun-Kai Wang, Xi Zeng, Zi-Jian Chen, Surat Hongsibsong, Wei Huang, Hong-Tao Lei, Yuan-Ming Sun and Zhen-Lin Xu *

* Correspondence: jallent@163.com

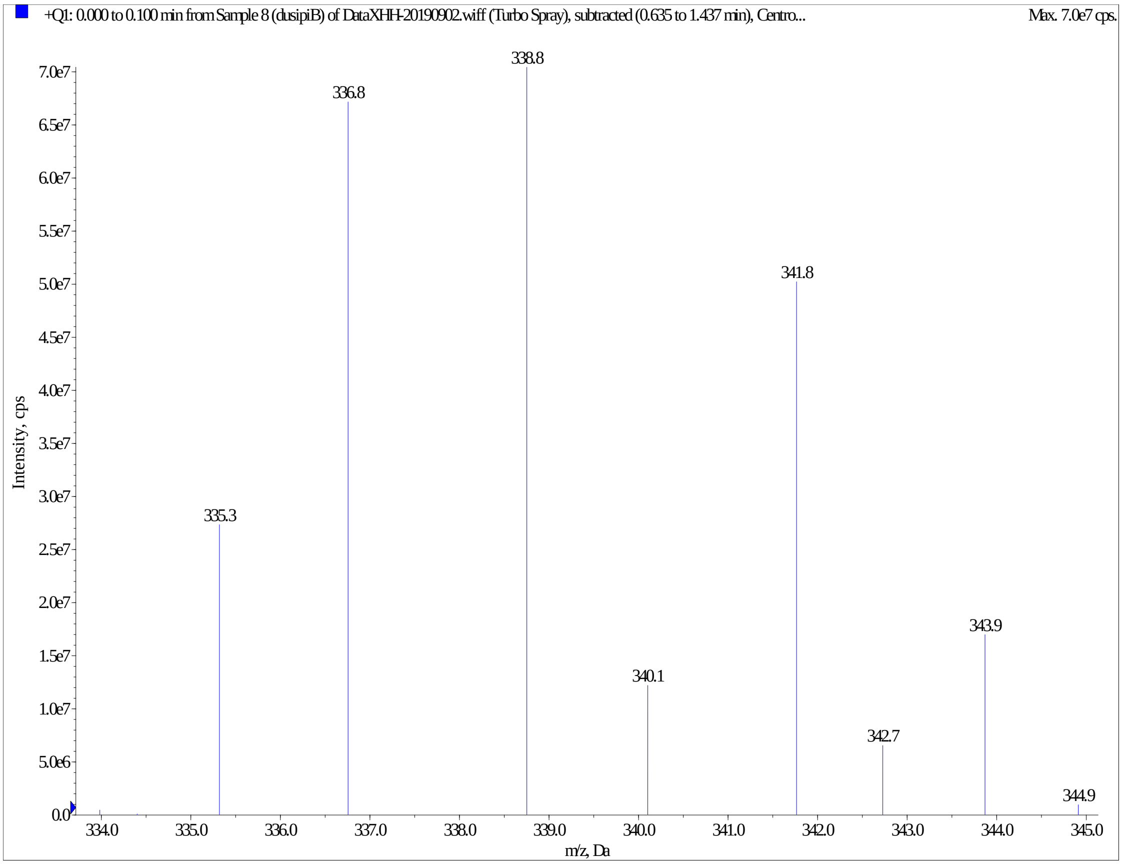

Figure S1. ESI-MS spectrum (positive) of CPS-H_1.

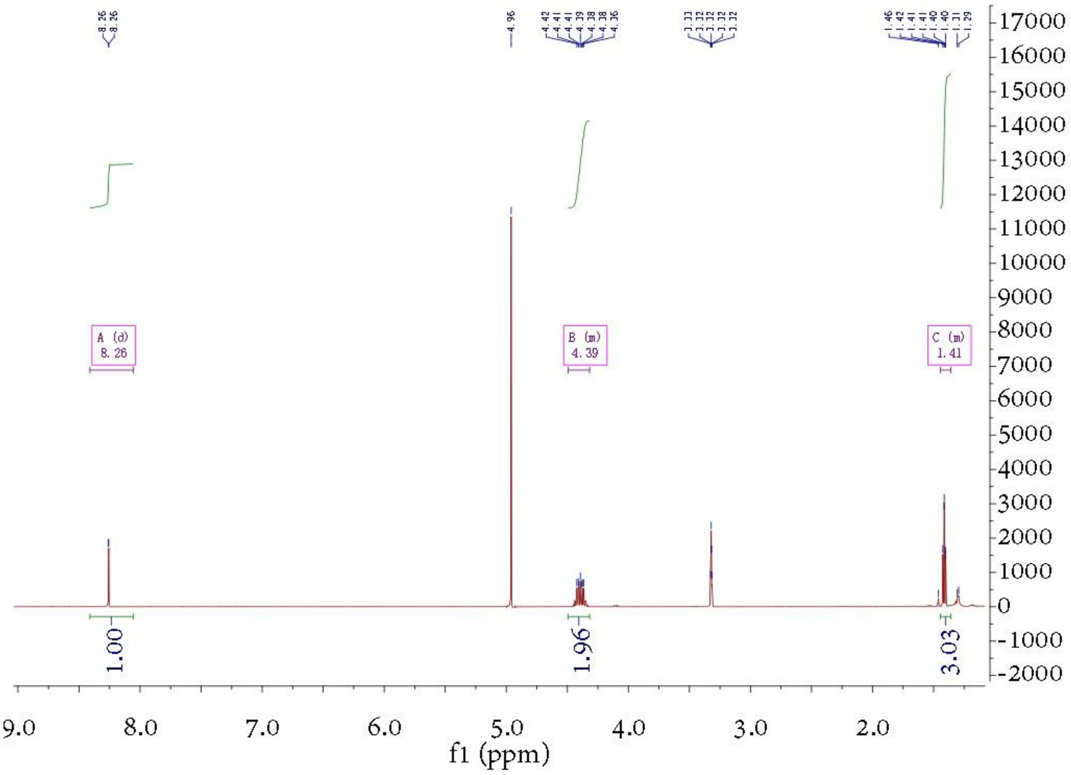

Figure S2. ^{1}H NMR spectrum of CPS-H_1.

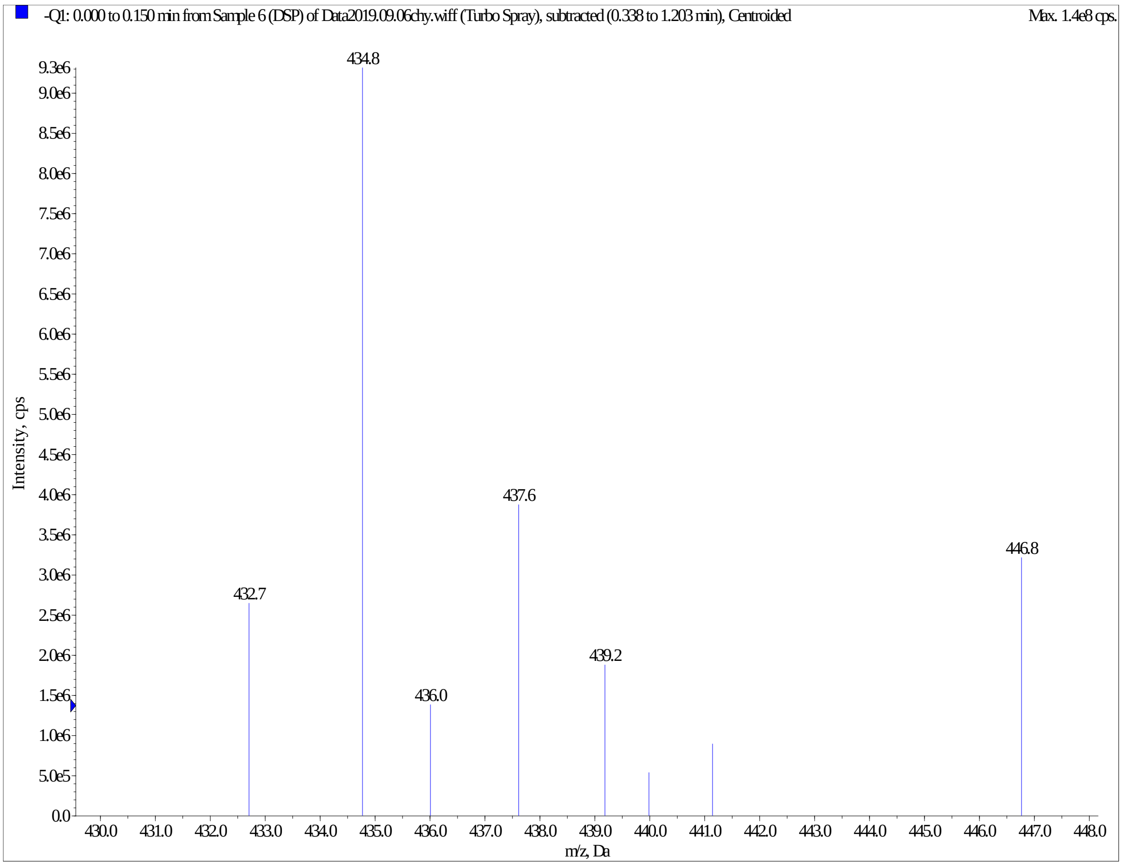

Figure S3. ESI-MS spectrum (negative) of CPS-H_2.

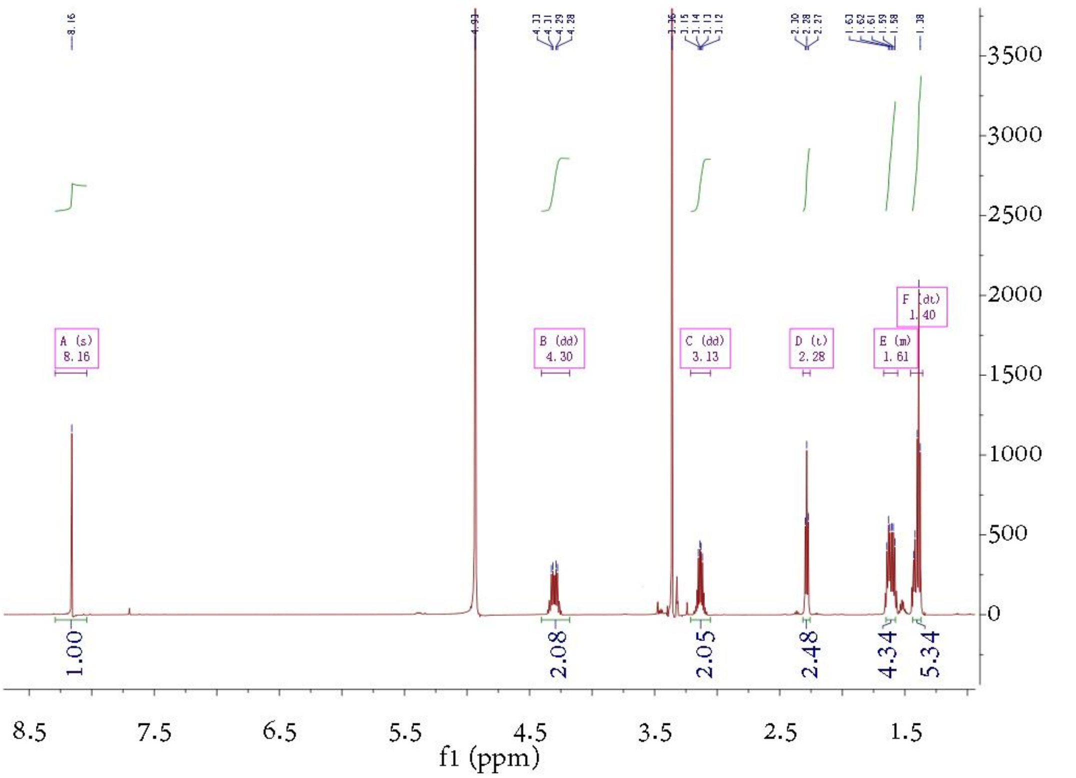

Figure S4. ^{1}H NMR spectrum of CPS-H_2.

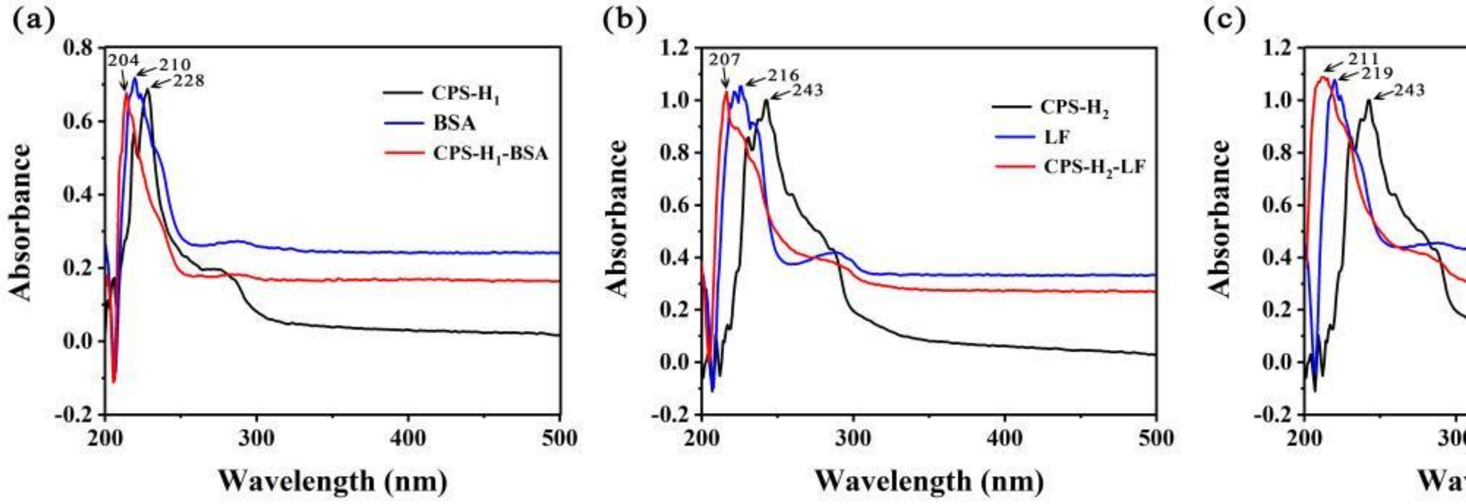

Figure S5. The UV wavelength scanning spectra of artificial antigens. **(a)** CPS-H_1-BSA. **(b)** CPS-H_2-LF. **(c)** CPS-H_2-BSA

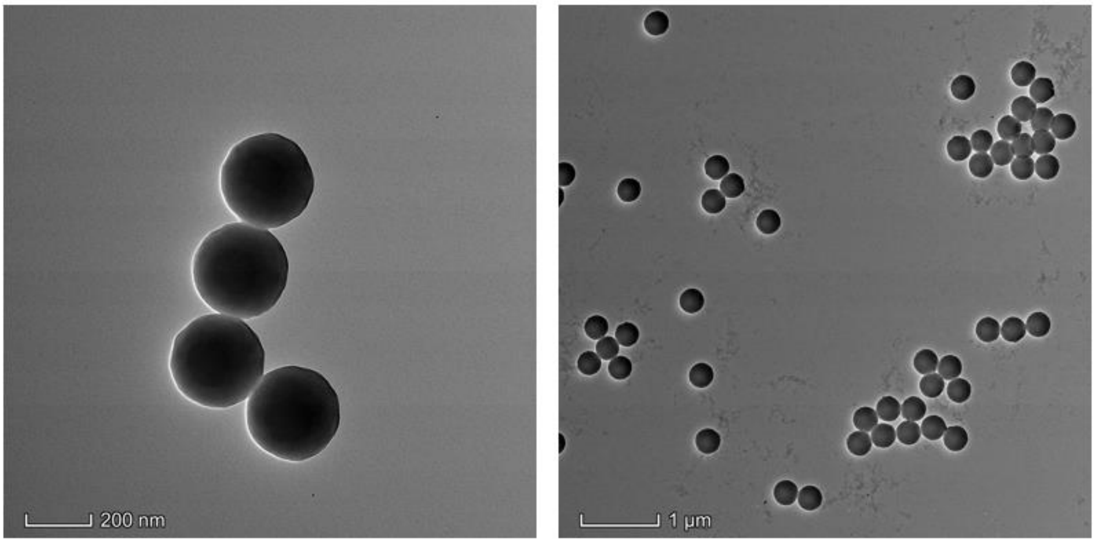

Figure S6. The electron microscope scan of fluorescence microspheres

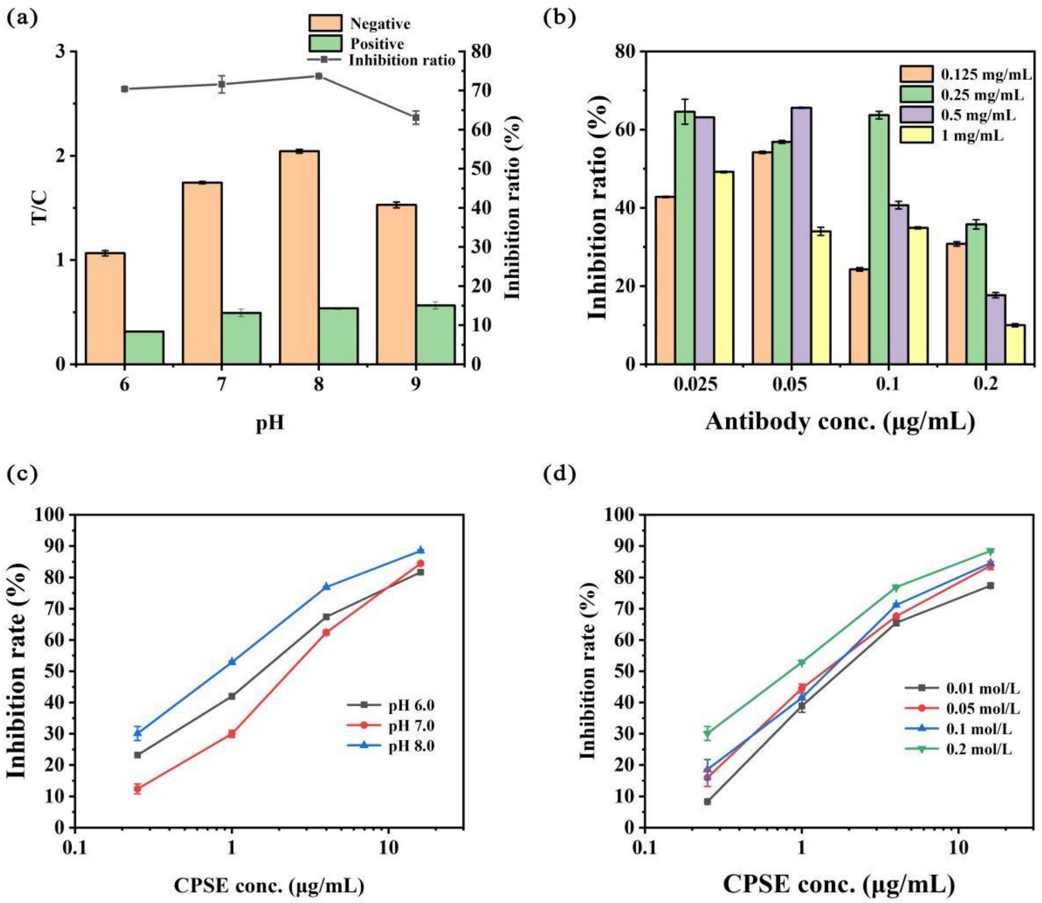

Figure S7. The signal intensity and inhibition results of (a) The labeling pH value; (b) The concentrations of antigen and antibody; (c-d) The pH and ion concentration of the CPSE standard buffer. The spiked target concentrations of the inhibitory effect in Fig. S7 (a-d) were 1 μg/mL.

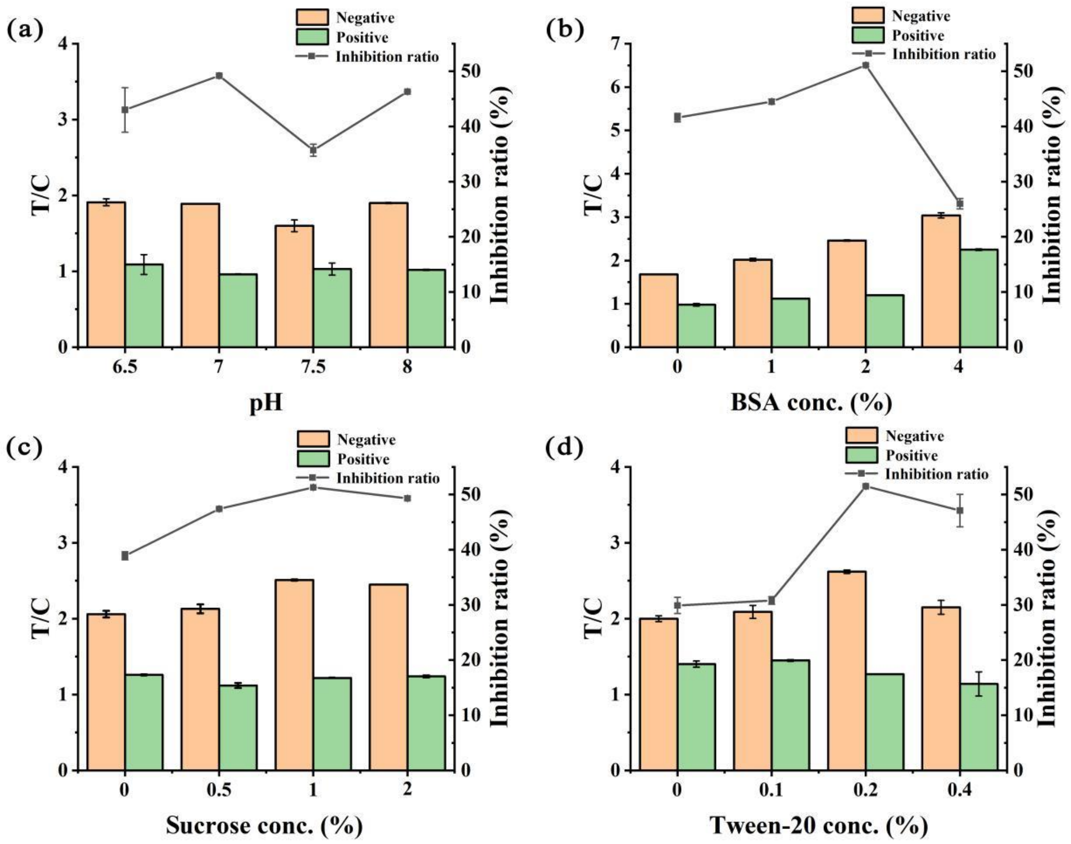

Figure S8. The signal intensity and inhibition results of fluorescence probe dilution (a) pH; (b) BSA concentration; (c) Sucrose concentration; (d) Tween-20 concentration. The spiked target concentrations of the inhibitory effect were 1 μg/mL.

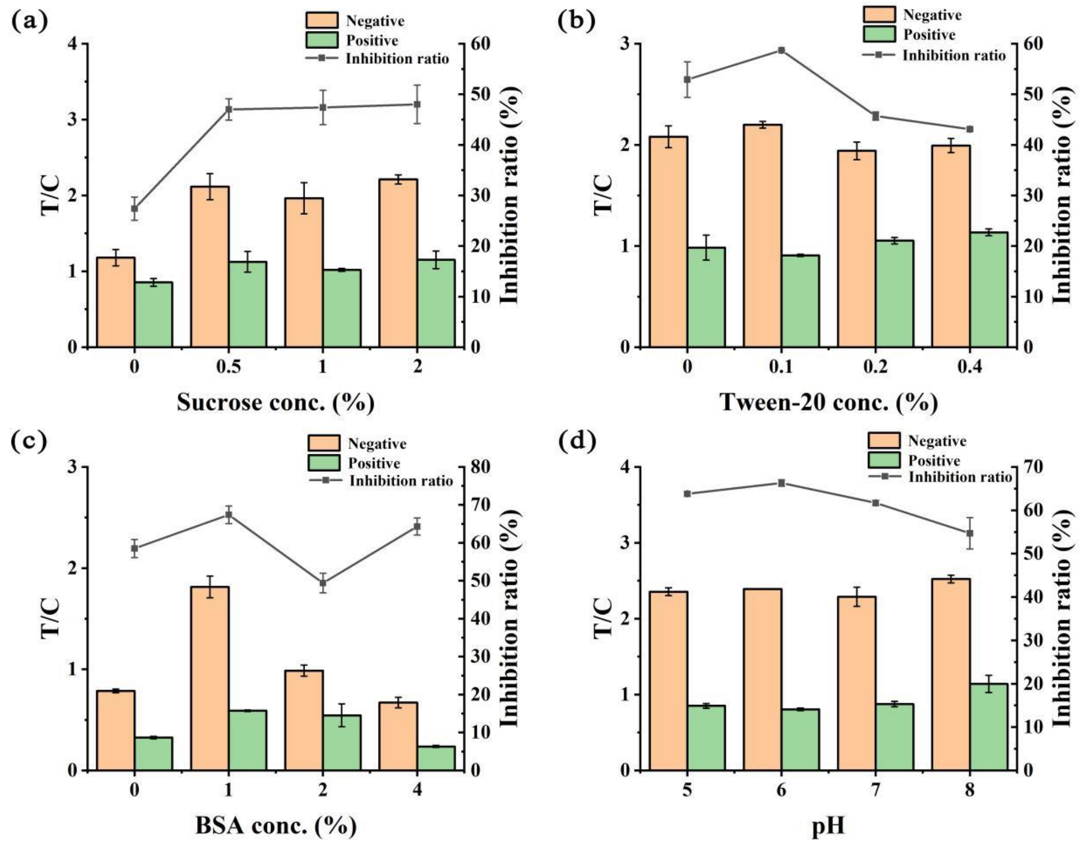

Figure S9. The signal intensity and inhibition results of sample pad pretreatment solution (a) Sucrose concentration; (b) Tween-20 concentration; (c) BSA concentration; (d) pH. The spiked target concentrations of the inhibitory effect were 1 μg/mL.

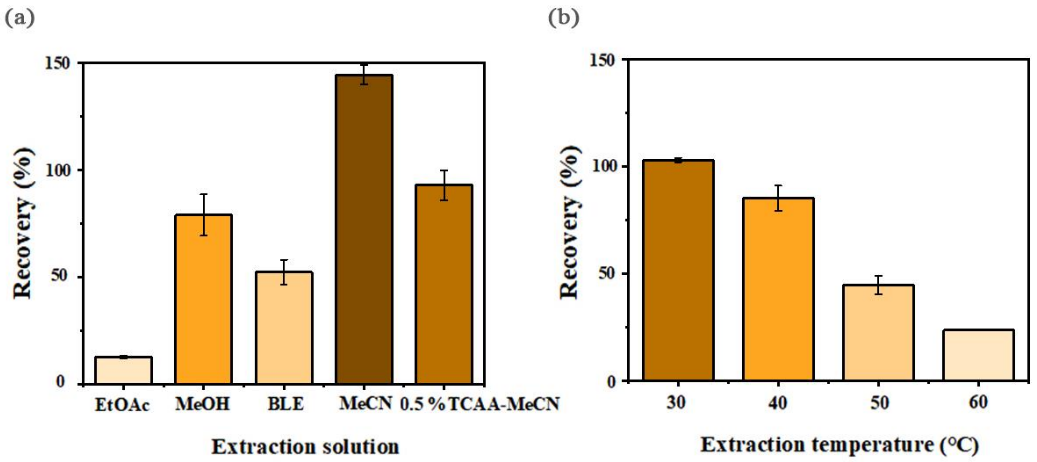

Figure S10. Optimization of sample pretreatment conditions (n=3). **(a)** Extraction solution. **(b)** Extraction temperature

Table S1. Characterization of the mouse antiserum against free CPSE.

Immunogen ($CPS\text{-}H_2\text{-}LF$)						
	Mouse 1		Mouse 2		Mouse 3	
Coating antigen	Titer[a] (×10^3)	Inhibition[b] (%)	Titer (×10^3)	Inhibition (%)	Titer (×10^3)	Inhibition (%)
$CPS\text{-}H_1\text{-}BSA$	1	43	8	64	8	50
$CPS\text{-}H_2\text{-}BSA$	16	2	16	5	16	ND[c]

[a]Titer is defined as dilution factor of antiserum with the absorbance at 450 nm being situated at about 1.0-1.5 at coating concentration of 1 μg/mL.

[b]Percentage inhibition was expressed as follow: inhibition (%)=[1-(B/B_0)]×100. B_0 was mean absorbance of the wells in the absence of competitor. B was mean absorbance of the wells in the presence of certain concentration of competitor.

[c]ND, no detected.

Table S2. Stability of fluorescent liquid (n=3).

Time	4 ℃			37 ℃		
(days)	T[a]	C[b]	T/C	T	C	T/C
2	6716	2331	2.881	6790	2362	2.875
4	6762	2435	2.777	6894	2510	2.747
6	6708	2410	2.783	6709	2496	2.688
8	6874	2608	2.636	6745	2577	2.617
10	6766	2334	2.899	6716	2331	2.881

[a]T was mean the fluorescence signal intensity of T line.

[b]C was mean the fluorescence signal intensity of C line.

Table S3. Stability of FICA (n=3).

Time	37 ℃			45 ℃		
（days）	T[a]	C[b]	T/C	T	C	T/C
1	6525	2414	2.703	6606	2409	2.742

7	6654	2247	2.961	6566	2261	2.904
15	6795	2476	2.744	6452	2286	2.822

[a]T was mean the fluorescence signal intensity of T line.

[b]C was mean the fluorescence signal intensity of C line.

Table S4. Test results of precision of intra-assay (n=3).

Number	T[a]	C[b]	T/C
1	5938	1994	2.978
2	5891	1980	2.975
3	5894	2131	2.766
4	6034	2190	2.883
5	5968	2070	2.871
CV	8.9%	3.9%	3.4%

[a]T was mean the fluorescence signal intensity of T line.

[b]C was mean the fluorescence signal intensity of C line.

Table S5. Test results of precision of inter-assay (n=3).

Number	T[a]	C[b]	T/C
1	5924	1865	3.176

2	5874	1869	3.142
3	6058	1981	3.058
4	5907	1914	3.086
5	5995	1887	3.177
CV	1.1%	2.5%	1.5%

[a]T was mean the fluorescence signal intensity of T line.

[b]C was mean the fluorescence signal intensity of C line.
